# Commentary: Healthy Eating Index-2020 and bowel habits: a cross-sectional analysis of NHANES

**DOI:** 10.3389/fnut.2025.1678925

**Published:** 2025-11-13

**Authors:** Amna Manahil, Ehtisham Haider, Maryam Waqar, Noor Un Nisa Irshad, Mohammed Hammad Jaber Amin

**Affiliations:** 1 Liaquat College of Medicine and Dentistry, Karachi, Pakistan; 2 Faisalabad Medical University, Faisalabad, Pakistan; 3 Institute of Business and Health Management, Dow University of Health Sciences, Karachi, Pakistan; 4 Jinnah Sindh Medical University, Karachi, Pakistan; 5 Al Zaiem Al Azhari University, Khartoum, Sudan

**Keywords:** Healthy Eating Index-2020, bowel habits, NHANES, cross-sectional analysis, nutrition

We congratulate the author Chen et al. (1) for presenting an insightful study exploring the link between Healthy Eating Index-2020 (HEI-2020) scores and bowel habits using NHANES data. Notably, higher HEI-2020 scores were related to considerably lower risks of constipation and accidental bowel leakage. Unrefined grains and unsaturated fat consumption emerged as key protective factors. While the cross-sectional design limits causal inference, this study offers valuable insights and a strong foundation for future prospective studies exploring diet-based interventions to enhance gastrointestinal health.

One important limitation is the omission of medication-related confounding. Several commonly prescribed drug classes, for example, opioids, certain antidepressants and anticholinergics, iron supplements, and calcium-channel blockers, are well-known to slow intestinal transit and increase constipation risk (2). Their use correlates with age, comorbidity burden, and socioeconomic factors that are themselves associated with diet quality and HEI measures (3). NHANES includes a prescription-medication module for the 2005–2010 cycles that can be used to identify therapeutic classes or medication counts (4). Because Chen et al. did not include medication exposures as covariates in their multivariable models (1), residual confounding by pharmacologic effects is plausible and could contribute to the observed HEI–constipation associations. We therefore suggest the authors (a) report the prevalence of recent use of opioids and other constipating medications in the analytic sample, (b) include prescription-medication variables (for example, opioid use, medication count, or therapeutic-class indicators from the NHANES RX files) as covariates, or (c) perform sensitivity analyses excluding users of known constipating drugs. These steps would help to determine whether the reported diet–bowel habit associations are robust to adjustment for pharmacologic influences.

Chen et al. appropriately noted the cross-sectional nature of their analysis (1), but we emphasize the risk of temporal bias (reverse causality) because of the timing of exposure and outcome measurement. Dietary exposure was derived from two non-consecutive 24-h recalls (HEI averaged from DR1 and DR2), whereas bowel symptoms were queried over the prior 30 days; this mismatch makes it plausible that symptoms preceded and modified short-term intake (for example, increased fiber intake in response to constipation), producing reverse causation or temporally biased associations. Yuan et al. describe how misaligned exposure/outcome windows may bias effect estimates (5), and Savitz and Wellenius discuss limits on causal inference from cross-sectional studies (6). We therefore recommend that Chen et al. explicitly state exposure/outcome windows in the Methods and add a brief discussion naming reverse causality as a plausible alternative explanation; prospective or longitudinal measurement would help support causal claims.

Chen et al. began with the NHANES sample and, after exclusions, arrived at the final analytic N (1). Specifically, the study started with 31,034 NHANES participants; participants with missing Bowel Health Questionnaire or HEI-2020 data (*n* = 18,318) and those with inflammatory bowel disease or colorectal cancer (*n* = 129) were excluded, resulting in a final analytic sample of 11,590. Excluding 19,444 participants (approximately 62.7% of the initial sample) is substantial and raises selection-bias concerns. Although the authors describe missing data handling, they do not present diagnostics comparing included vs. excluded participants or sensitivity analyses using principled methods. We recommend (a) a table comparing excluded vs. included participants on key sociodemographic and clinical variables, and (b) application of multiple imputation (or combined MI/IPW) with comparison to complete-case results, as multiple imputation can reduce bias relative to listwise deletion in many practical settings (7).

Outcome ascertainment relied on self-report questionnaires and the Bristol Stool Form Scale (BSFS) without clinician adjudication, a limitation Chen et al. acknowledge (1). We reiterate this because studies show only modest concordance between self-reported BSFS and objective stool consistency measures (for example, stool water content) in some clinical groups, implying potential misclassification of constipation/diarrhea/incontinence (8). Where feasible, validation against clinical records or objective stool measures, or sensitivity analyses using alternative definitions, would strengthen inference.

Chen et al. appropriately restrict inference to the U.S. non-institutionalized adult population (1), and we echo this caution. HEI-based associations observed in NHANES may not generalize to countries with different food availability, cultural dietary patterns, or healthcare systems (for example, Pakistan). International approaches to dietary pattern construction differ, and cross-population replication is therefore important before extrapolating policy or clinical implications (9). Replication in regionally representative cohorts or targeted intervention studies would clarify external validity.

Chen et al. note the absence of gut microbiome and other objective biomarkers as a limitation (1). We concur and add that targeted biomarkers (for example, fecal microbiome sequencing, fecal SCFAs, fecal calprotectin, and systemic inflammatory markers) or metabolomic profiling would allow mechanistic and mediation analyses to test whether diet-bowel associations operate via microbial or inflammatory pathways. Recent reviews of metagenomic studies emphasize the value of combined biomarker approaches in diet–microbiota research (10).

The authors deserve appreciation for leveraging a nationally representative dataset to address an issue that is current and clinically important. Despite some limitations, their work greatly improves our knowledge of the relation between dietary quality and bowel health and establishes a solid foundation for future research.
